# In Vitro and In Silico Analysis of Entrainment Characterization in Injection Jet-Assisted Fontan Circulation

**DOI:** 10.3390/bioengineering12050555

**Published:** 2025-05-21

**Authors:** Arka Das, Ray O. Prather, Anthony Damon, Michael Farias, Alain Kassab, Eduardo Divo, William DeCampli

**Affiliations:** 1 Department of Mechanical Engineering, Embry-Riddle Aeronautical University, Daytona Beach, FL 32114, USA; prather3@erau.edu (R.O.P.); damona1@my.erau.edu (A.D.); divoe@erau.edu (E.D.); 2 Department of Mechanical and Aerospace Engineering, University of Central Florida, Orlando, FL 32816, USA; alain.kassab@ucf.edu; 3 Department of Cardiology, Boston Children’s Hospital, Boston, MA 02115, USA; michael.farias@cardio.chboston.org; 4 The Heart Institute at Orlando Health Arnold Palmer Hospital for Children, Orlando, FL 32806, USA; william.decampli@orlandohealth.com; 5 Department of Clinical Sciences, College of Medicine, University of Central Florida, Orlando, FL 32816, USA

**Keywords:** congenital heart defect, single ventricle defect, Fontan physiology, in vitro modeling, in silico modeling, entrainment characteristics

## Abstract

Fontan circulation is a fragile system in which imperfections at any of multiple levels may compromise the quality of life, produce secondary pathophysiology, and shorten life span. Increased inferior vena caval pressure itself may play a role in “Fontan failure”. This study describes a mock flow loop model (MFL) designed to quantitatively estimate pulmonary flow entrainment induced by continuous and pulsed flow injections. A patient generic 3D-printed phantom model of the total cavopulmonary connection (TCPC) with average dimensions matching those of a 2–4-year-old patient was inserted in an MFL derived from a reduced lumped parameter model (LPM) representing cardiovascular circulation. The LPM comprises four 2-element Windkessel compartments (compliance and resistance), approximating the upper and lower systemic circulations and the right and left pulmonary circulations. The prescribed cardiac output is about 2.3 L/min for a body surface area of $0.675{\;m}^{2}$. The injections originate from an external pump through a 7–9 fr catheter, following a strict protocol suggested by the clinical team, featuring a variation in injection rate (flow rate), injection volume, and injection modality (continuous or pulsed). The key measurements in this study are the flow rates sampled at the distal pulmonary arteries, as well as at the upper and lower body boundaries. These measurements were then used to calculate effective entrainment as the difference between the measured and expected flow rates, as well as jet relaxation (rise and fall time of injection). The results show that for continuous or pulsed injections, varying the total volume injected has no significant influence on the entrainment rate across all injection rates. On the other hand, for both injection modalities, increasing the injection rate results in a reduction in entrainment that is consistent across all injected volumes. This study demonstrates the effectiveness of a high-speed injection jet entraining a slow co-flow while determining the potential for fluid buildup, which could ultimately cause an increase in caval pressure. To avoid the increase in caval pressure due to mass accumulation, we added a fenestration to our proposed injection jet shunt-assisted Fontan models. It was found that for a set of well-defined parameters, the jet not only can be beneficial to the local flow, but any adverse effect can be obviated by careful tuning. These results were also cross-validated with similar in silico findings.

## 1. Introduction

**Figure 1:**
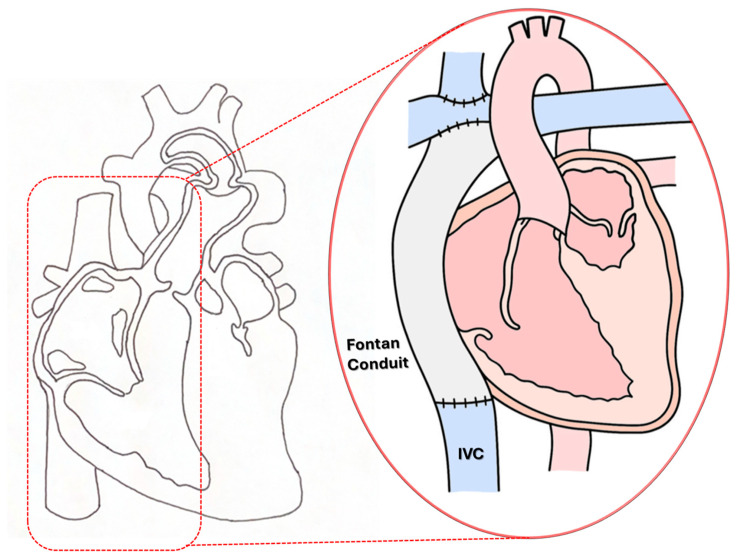
Schematic illustration of the third-stage “Fontan Kreutzer” palliative procedure for a single ventricle (SV) anomaly.

Normal biventricular circulation has two distinct pumping chambers known as ventricles. The left ventricle is responsible for delivering oxygen-rich blood to the entire body (referred to as “systemic circulation”). Meanwhile, the right ventricle transports blood from the body to the lungs (known as “pulmonary circulation”). Approximately 1 in 3841 babies is born with hypoplastic left heart syndrome (HLHS) [1,2,3,4,5]. These patients cannot survive without a series of palliative surgeries designed to ensure sufficient blood flow to both systemic and pulmonary circulations [3]. The Fontan procedure represents the final step in the standard palliative surgical approach for neonates born with a single ventricular defect [6,7,8]. During this procedure, the inferior vena cava (IVC) is connected to the pulmonary artery (PA) using either an extracardiac expanded polytetrafluoroethylene (ePTFE) graft or an intra-atrial lateral tunnel baffle. This creates a total cavopulmonary connection (TCPC), allowing systemic venous return to flow directly to the PA [7,8] as shown in Figure 1.

Although early mortality rates following the Fontan procedure have significantly decreased, long-term health issues and mortality still pose significant risks [8,9,10,11,12,13,14,15,16,17]. These complications arise mainly from the limitations of univentricular Fontan circulation, which does not include a functional subpulmonary ventricle, leading to non-pulsatile venous flow to the lungs lacking momentum and power [18,19,20,21,22,23]. A recent multi-institutional study from the Australian and New Zealand Fontan Registry, which examined 683 adult Fontan patients, revealed that by the age of 40, the mortality rate had reached 20% [24]. Additionally, only 53% of the patients remained free from heart failure symptoms, and only 41% avoided serious adverse events [24]. These data align with a broader range of findings in the literature [8,9,10,11]. Notably, approximately half of the mortality and significant morbidity cases can be linked to the dysfunction of the Fontan circulation, with the mechanisms of failure being complex and not yet fully elucidated [25,26,27].

Numerous enhancements to the Fontan pathway have been explored, focusing on the placement, shape, and dimensions of the inferior cavopulmonary connection [28,29,30,31]. A range of computational and experimental models have been developed to examine the hemodynamic effects of this surgical approach [32,33,34,35,36,37,38,39,40,41]. To investigate mechanical cardiac support in Fontan physiology, benchtop MFLs have been designed to replicate realistic circulatory conditions, enabling patient-specific simulations for staged Fontan procedures and various in vitro trials. Trusty et al. [29], for example, utilized MFLs to assess the effectiveness of PediMag and CentriMag devices in supporting compromised Fontan circulation. Similarly, Yamada et al. [42] designed an MFL to test a pulmonary assist device, incorporating shape memory alloy fibers to enhance flow within total cavopulmonary connections. MFLs have also proved valuable in evaluating different cardiopulmonary support configurations. Dur et al. [43] examined Medos and Pediaflow Gen-0 pediatric ventricular assist devices (VADs) in Fontan settings, stressing the importance of patient-specific VAD adjustments. Another MFL setup incorporated a von Karman viscous impeller pump, providing mechanical support to optimize cavopulmonary flow, overcoming obstructions, and maintaining target pressure levels [44,45]. Collectively, these studies highlight the promising potential of passive Fontan-specific devices to address the unique hemodynamic challenges associated with single-ventricle physiology, paving the way for enhanced management strategies in this patient population.

In our earlier research, we investigated a novel approach to energize Fontan circulation through the implementation of an injection jet shunt (IJS) [38,39,40,41,46]. This shunt utilizes flow derived from the aorta, which is then directed into the Fontan conduit, aligning it with the co-flow of the inferior vena cava (IVC) [38,47,48]. The basic concept involves the injection of a high-velocity fluid jet along the flow direction to entrain the ambient flow, resulting in a reduction of upstream (proximal) pressure and enhancement of downstream (distal) flow [34,38,39,40,41,46,47,48,49]. Distinct from traditional mechanical pumps, this passive pumping mechanism remains entirely intra-corporeal, devoid of moving components, and is driven by the native functioning ventricle. Through extensive parameter evaluations, we established that the IJS could effectively reduce central venous pressure by approximately 3–4 mmHg, while maintaining a clinically tolerable increase in ventricular preload and an acceptable decline in arterial oxygen saturation [40]. Our previously reported in silico findings show that a surgically implementable IJS-assisted total cavopulmonary connection (TCPC) has the potential to decrease Fontan pressure while tapping the available ventricle’s reserve capacity, maintaining a clinically acceptable ratio of total cardiac output (CO) to systemic flow (Qs). 

The driving principle of the injection jet shunt is the entrainment effect, which effectively transfers momentum from the high-velocity jet flow to the low-velocity co-flow [50,51,52,53,54,55,56,57]. Entrainment results from the strong shearing at the interface of the two interacting flow regimes. The momentum transfer is heavily influenced by the residual pulsatility and the velocity ratio, which is constrained by the shunt size and injection flow rate [58,59,60,61,62,63]. While the Fontan circulation is weakly pulsatile, the flow shunted from systemic circulation is strongly pulsatile. It is expected that pulsatility will significantly influence the entrainment profile of the IJS. Therefore, it is important to quantify the IJS efficacy characteristics in terms of effective entrainment and transient dynamics during injection.

In this study, in vitro and in silico techniques were used to investigate the IJS entrainment characteristics in Fontan circulation. A specialized CFD model was constructed to isolate and highlight the IJS contribution to the Fontan flow field. The model obviates the need for the complex and computationally expensive multiscale framework typically required to observe the IJS effect, by implementing the specialized boundary conditions. For conducting the in vitro study, a reliable benchtop configuration was required to replicate a physiologically consistent Fontan flow field. To achieve this, a dynamically scaled MFL was developed to incorporate 3D-printed phantoms of Fontan TCPC with the injection port. Initially, baseline Fontan physiology was simulated experimentally, followed by targeted injections into the RPA branch based on an experimental protocol developed by our clinical collaborators from Orlando Health. This experimental study investigates the effects of various injection parameters on Fontan hemodynamics, including (i) injection volume, (ii) rate, and (iii) different modes: continuous or pulsed.

## 2. Materials and Methods

To isolate and characterize the intrinsic entrainment dynamics of the injection jet shunt (IJS), this study is conducted using a non-fenestrated Fontan model. While a fenestration is present in the majority of clinical Fontan anatomies, its role in the IJS-assisted context is not to directly relieve pressure, but rather to accommodate excess volume buildup. Including fenestration at this stage would introduce overlapping flow pathways that could obscure the standalone behavior of the IJS jet. Our aim in this study is to create a controlled testbed for examining the fluid mechanics of entrainment in isolation, without the confounding influence of secondary shunt flows. This foundational analysis provides insight into how the IJS independently contributes to pressure reduction via an entrainment mechanism, and it forms the basis for our ongoing investigations involving more complete models that incorporate fenestrated Fontan geometries of 2–5-year-old patients. These follow-up studies will address the coupled dynamics of IJS and fenestration, which together form a surgically feasible and physiologically realistic construct for the failing Fontan circulation, and will be reported elsewhere.

### 2.1. Anatomical Model

#### 2.1.1. In Silico Phantom

**Figure 2:**
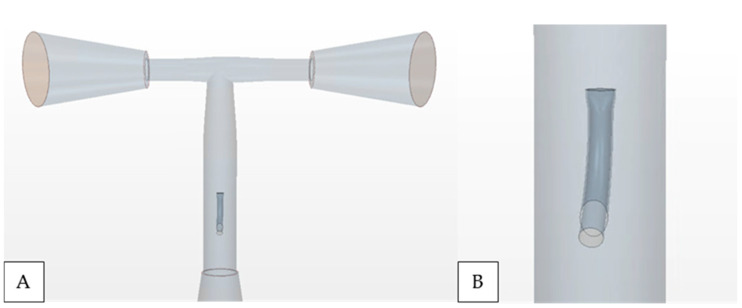
(**A**) A simplified isolated T-junction TCPC morphology and (**B**) elliptical nozzle IJS in the Fontan conduit implemented along the centerline.

The purpose of this analysis is to investigate the co-flow dynamics in a simplified model of patient-generic geometry. The anatomical dimensions are derived from measurements obtained from 2–5-year-old patients with an approximate $BSA\;\;\mathrm{of}\;0.675\;m^{2}$, and for simplicity, the model only accounts for the inferior vena cava (IVC), IJS, and left/right pulmonary arteries (LPA and RPA). Figure 2A displays sample geometry replicating the morphology of an extracardiac Fontan, including the insertion of the IJS in the Fontan conduit (Figure 2B). In our previous study, we focused on quantifying the outcomes of several geometrical parameters, such as IJS configuration, TCPC morphology, fenestration size, and IJS distance to the TCPC. In the current effort, we implement the geometry in Figure 1 with an elliptical 2 mm IJS with an aspect ratio of 3 and an approximate IJS-TCPC distance of 7 cm.

As mentioned earlier, this study only aims to quantify and qualify the jet entrainment effect on IVC pressure and flow while keeping all geometrical parameters constant.

*Mesh*

**Figure 3:**
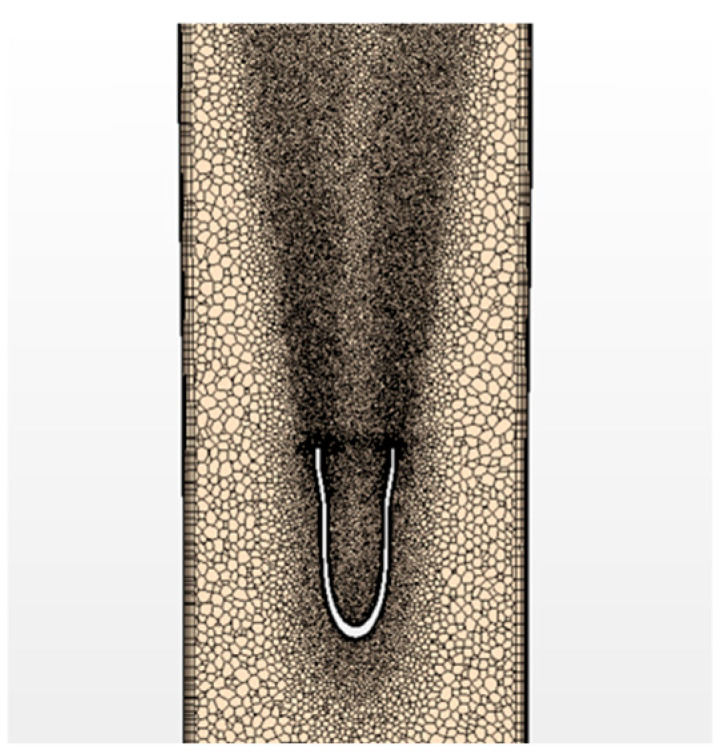
Mesh detail in the Fontan conduit near the IJS displaying IJS wall surface refinement and volumetric wake refinement.

Once the geometry was generated, it was imported into the CFD destination software (StarCCM+ (Siemens, Munich, Germany), https://plm.sw.siemens.com/en-US/simcenter/fluids-thermal-simulation/star-ccm/, accessed on 1 December 2024) to be meshed and to carry out detailed computational fluid dynamics analyses (CFD). Following Roche index calculations for 3 degrees of refinement confirming a converged mesh, the final mesh features polyhedral cells with a base size of about 0.6 mm, resulting in a total cell count of ~3 M cells. Polyhedral cells were chosen since they are well suited for irregular morphologies, allowing a high degree of accuracy with a lower mesh count [64,65]. At all wall boundaries, there are 5 prism layers scaled to the size of the conduit. To capture the transitional characteristics of the flow induced by the jet—from laminar during diastole to turbulent during systole [40]—significant mesh refinement was applied in the axial direction along the IVC downstream of the IJS nozzle inlet. Flow features typical of turbulent flows, such as eddies and vortices, were resolved using two spatial refinements: (1) surface mesh refinement applied to the inner and outer IJS surface to accurately capture flow interactions near the IJS, and (2) cone-like volume refinement in the wake of the nozzle, spreading at a 7° angle up to the TCPC (Figure 3). Refinement results in cell sizes ranging from 36 to 72 μm, a significant refinement with respect to previous studies [40].

#### 2.1.2. In Vitro Phantom

**Figure 4:**
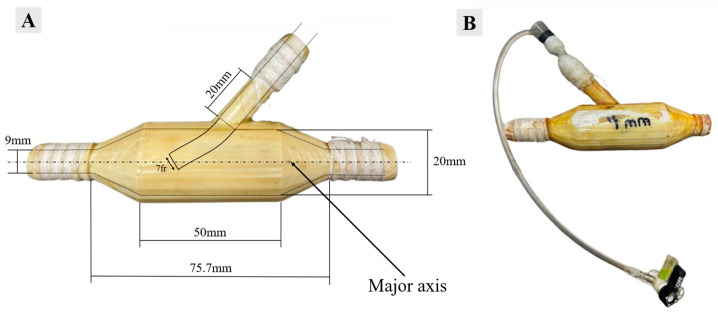
(**A**) RPA injection shunt dimensions and (**B**) RPA injection shunt with a 7 fr catheter inserted.

In the experimental study, a patient generic total cavopulmonary connection (TCPC) phantom was developed using SolidWorks (Dassault Systèmes, version 2022, France), incorporating average dimensions representative of 2–5-year-old Fontan patients. The in vitro study investigates the entrainment effects resulting from fluid injections into the right pulmonary artery (RPA), utilizing a custom-designed 3D-printed injection shunt, as illustrated in Figure 4A. The design of the injection shunt features an angled port leading into the conduit, with an inner diameter of 4 mm oriented along the shunt major axis. To facilitate integration with the MFL, the experimental phantom was equipped with 5/8″ barbed connectors. The shunt port allows for the accommodation of a 7 Fr catheter within the confines of the 3D-printed phantom, as depicted in Figure 4B. The dimensions for the TCPC were established based on the anatomical measurements of the RPA, LPA, SVC, and IVC, with diameters of 6 mm, 9 mm, 12 mm, and 18 mm, respectively. A complete list of acronyms and abbreviations can be found in Appendix A Table A1. As explained in our previous report [39], the TCPC assembly was created using a two-part fabrication process in this experimental study. Both components were constructed using acrylonitrile butadiene styrene (ABS) via 3D printing technology. After manufacturing, the top and bottom halves were securely bonded using adhesive sealants and epoxies to prevent any leakage during experimental testing. The terminal ends of the TCPC were coupled to the conduits with clamps when interfaced with the MFL to ensure stability and accuracy throughout the experimental procedure.

### 2.2. In Vitro Lumped Parameter Model

The lumped parameter model serves as an electric circuit analogy to mimic peripheral circulation within the human cardiovascular system. In this framework, blood flow in different cardiovascular segments is simplified and modeled using electrical components such as resistors, inductors, and capacitors, which represent vascular resistance, vascular inertance, and vascular compliance, respectively. In recent decades, various LPM configurations have been designed to experimentally replicate different reconstructed cardiovascular anatomies. The primary aim of these LPM setups is to link the fluid dynamics observed in a 3D phantom with the hemodynamics of peripheral circulation.

In the in vitro study, a reduced-order LPM was developed specifically for constructing the MFL test rigs necessary for conducting experiments. This setup was tuned to ensure accurate control of overall resistance and compliance across each branch of the LPM.

Notably, Ni et al. [34] and Prather et al. [40] created a comprehensive full-scale LPM representing Fontan circulation, which includes compartments for the carotid, subclavian, right pulmonary, left pulmonary, lower body, Fontan TCPC, and coronary flow branches. Each branch, designed with both arterial and venous beds utilizes a three-element Windkessel compartment. However, as discussed by Das et al. [39], implementing a laboratory benchtop realization based on this full-scale LPM model proves unfeasible. Consequently, the Fontan circulation MFL setup is based on a reduced-order LPM derived from the works of Ni et al. and Prather et al. 

**Figure 5:**
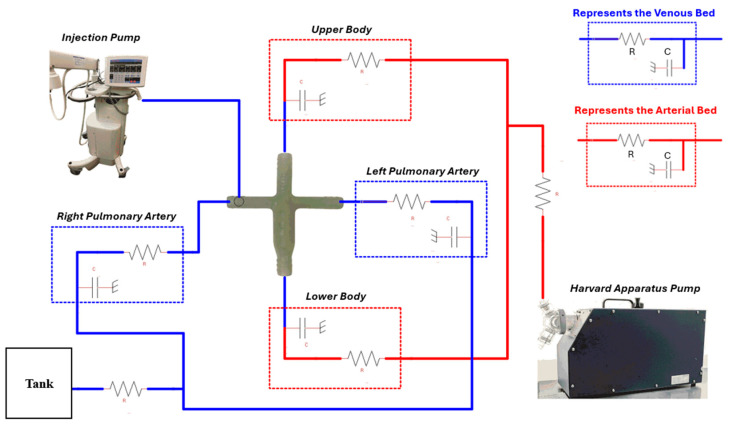
Lumped parameter model for the benchtop setup.

This in vitro study leverages the same reduced-order LPM framework that has been meticulously detailed by Das et al. [39]. The hydrodynamic metrics assessed in this research encompass flow rates and pressures measured at the systemic and pulmonary junctions connected to the Fontan TCPC conduits. This reduced-order LPM, utilized for the MFL, comprises four compartments designed to accurately simulate Fontan dynamics under pulsatile conditions. The vascular compliance values for the in vitro experiments are derived from physiological data corresponding to a 2–5-year-old patient with a body surface area (BSA) of 0.675 m^2^, catering to benchtop trials. Each compartment in this in vitro model was structured using two components—resistance and capacitance, as illustrated in Figure 5. The in vitro LPM features two systemic compartments, representing the upper and lower systemic circulations, alongside two pulmonary compartments for the right and left pulmonary circulations, respectively. A detailed table outlining the nomenclature and location of the lumped parameter model (LPM) elements used in each compartment of the in vitro study is provided in Appendix A Table A2. Each branch within the MFL was equipped with a device that corresponds to a specific circuit element of the reduced LPM.

### 2.3. In Silico Flow Modeling

The commercial CFD software StarCCM+ (Siemens, Munich, Germany) provides an integrated meshing and flow solver environment well suited for detailed computational flow analysis. The flow solver is designed to resolve the time-dependent, incompressible Navier–Stokes equations, where Equation (1) enforces mass conservation through the continuity equation, and Equation (2) describes momentum conservation.

(1) $$\nabla \cdot \vec{V}=0$$

(2) $$\rho \frac{\partial \vec{V}}{\partial t}+\rho \left(\vec{V}\cdot \nabla \right)\vec{V}=-\nabla p+\nabla \cdot \sigma \;$$

Here, ρ is the density of blood, taken as 1060 kg/m^3^, V is the velocity field, p is the pressure, and $\sigma =\mu \left(\dot{\gamma }\right)\left[\nabla V+{\left(\nabla V\right)}^{T}\right]$ is the viscous stress tensor with a given relation for viscosity $\mu \left(\dot{\gamma }\right)$ as a function of shear rate $\dot{\gamma }$. The non-Newtonian rheology of blood was modeled as a Carreau–Yasuda fluid based on a simplified three-parameter model [66]. No fluid–solid interaction was accounted for; hence, the fluid domain walls were assumed to be rigid. The 3D pulsatile hemodynamics in the fluid domain are modeled as unsteady, turbulent, and incompressible flow. The IJS inlet boundary was assigned a mass flow rate, the IVC boundary was assigned a stagnation pressure of 18.005 mmHg (which generates a 0.0026 L/min flow rate), and the boundary conditions at the exits (RPA and LPA) were given a constant static pressure of 18 mmHg. These high pressures mimic the physiology of a failing Fontan. The IJS input time-dependent waveforms were obtained from the converged solutions obtained and detailed in previous studies implementing a tightly coupled CFD-LPM model [40]. To gain insight into the entrainment effect upstream of the IJS at the IVC boundary, a stagnation pressure nearly identical to the downstream outlet pressure was imposed to instantiate near-stagnant flow in the region. To impose an IVC flow waveform without implementing a mass flow or velocity inlet boundary condition, a volume force was applied in the cylindrical region upstream of the IJS near the IVC boundary. This volume force is time dependent, and following an initial calibration, it can be generated by volumetric interpolation with respect to a known IVC flow waveform. By applying a volume force to elicit IVC flow as opposed to a mass flow boundary condition, the flow entrainment resultant pressure drop at the IVC can be measured without complex coupling schemes, significantly reducing model complexity and computational expenses.

As observed in previous studies, the ambient flow and the jet have two separate flow regimes. At peak systole, the jet flow can be considered turbulent; in diastole, the same jet becomes laminar. To ensure that these dynamics features are tracked, the turbulent model of choice is large eddy simulation (LES) combined with the aforementioned grid refinement and a wall-adapted local eddy-viscosity (WALE) sub-grid model to compute flow below the cutoff length scale.

The model assumes unsteady conditions with time discretization, implementing a second-order implicit scheme with a multistep predictor-corrector backward differential formulation (BDF). The time step is dictated by the HR and the converged mesh’s average base size to meet the Courant number (Co) stability and time-accurate requirements (Co = 1), requiring a time step of ~0.003 s. Convection is modeled using a bounded central differencing scheme mediated by a blending function. The time-step convergence criteria for continuity, x-momentum, y-momentum, and z-momentum were set at 10^−3^.

### 2.4. Experimentation

**Figure 6:**
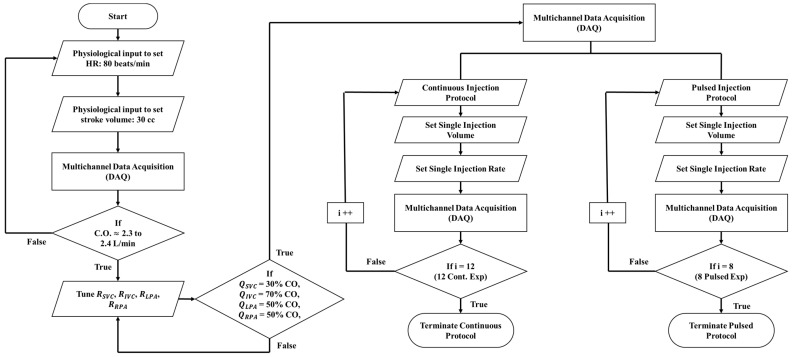
MFL tuning flowchart for continuous and pulsed injection protocols. Baseline Fontan conditions are established before proceeding with the desired injection sequence.

The MFL setup for the benchtop study includes the systemic and pulmonary circulation components and is strictly derived from the reduced LPM model discussed in Section 2.2. Each hemodynamic parameter (flow rate and pressure) in the LPM has a specified value derived from clinical measurements, and these values are realized in the MFL by replicating components in each circulation bed. Each circulation compartment contains an adjustable needle resistor valve to initialize flow splits and custom-built compliance chambers to replicate vascular compliance and pulsatility in the Fontan circulation. The key to achieving meaningful results in MFL setups depends highly on the accurate tuning of these individual components and the data acquisition process. A precise tuning process was followed to ensure that proper Fontan flow dynamics were initialized in the baseline case following the procedure shown in Figure 6. To establish the baseline condition, the cardiovascular waveform pump (Model 1421, Harvard Apparatus, Holliston, MA, USA) was calibrated to operate at 80 beats per minute (bpm) with a stroke volume of 30 cc, achieving a target Fontan cardiac output (CO) of approximately ~2.3–2.4 L/min. Following CO initialization, vascular resistances in each Windkessel compartment, i.e., $R_{SVC}$, $R_{IVC}$, $R_{LPA}$, and $R_{RPA}$, (Appendix A Table A1 and Table A2) are adjusted to control the flow ratios in both systemic and pulmonary circulation such that 30% of CO is circulated to the upper body and 70% of CO is circulated to the lower body. Returning venous flow was equally distributed to the right and left pulmonary circulation. Data acquisition cards NI DAQ 9361 and NI 9205 (National Instruments, Austin, TX, USA) were used in conjunction with in-house written LabVIEW (National Instruments, Austin, TX, USA) and Matlab (MathWorks, Natick, MA, USA) codes to acquire hemodynamic flow field responses at every LPM compartment.

**Figure 7:**
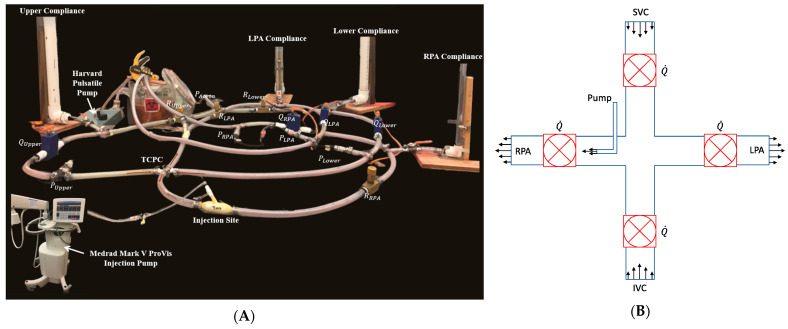
(**A**) Labeled mock flow loop setup depicting LPM compartments and injection site. (**B**) Schematic of in vitro TCPC section.

After setting the baseline flow ratios, we proceeded with entrainment investigations using a continuous and pulsed jet injection protocol designed by our clinical collaborators, as shown in Table 1. Specifically, the Medrad Mark V ProVis angiographic injector (Bayer AG, Leverkusen, Germany) provided by our clinical collaborators at Orlando Health was used to inject 12 continuous (long single injection) and 8 pulsed injections (short multiple injections) to the RPA injection site as shown in Figure 7A. After the multichannel data acquisition (DAQ) was engaged to capture the flow rate and pressure signals in each branch, the injection volume and rate were set as described in Figure 6. The Mark V ProVis pump uses a syringe injection method with a capacity ranging from 0–200 mL and injection rates from 0.3 to 50 mL/s. Depending on the experimental trial, the injection volume (in CC or mL) and speed (in cc/s or mL/s) was configured on the pump control interface. For pulsed injections, the pump speed was variably controlled for the duration of each injection sequence.

The flow rate of the injected fluid varied between 5 cc/s, 10 cc/s, 15 cc/s, and 20 cc/s for both the continuous and the pulsed injections. The total volume of the injected fluid depends on the number of bursts and the duration for which the jet is engaged. Hence, the short-pulsed injections consisted of multiple low-volume injections, while the long continuous injection was a single high-volume injection into the RPA. Table 1 includes the detailed injection protocol studied in this experiment for continuous and pulsatile runs.

#### 2.4.1. Post-Processing Scheme

**Figure 8:**
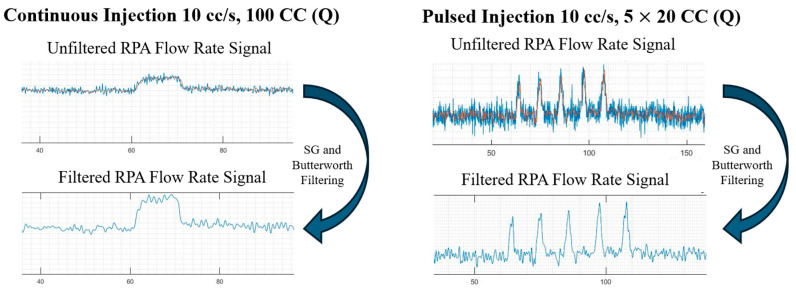
Filtering mechanism employed on continuous and pulsed injections.

During experimental trials, significant noise interference was observed in pressure and flow rate readings sampled at various locations, as shown in Figure 7B. Hence, a post-processing scheme involving Butterworth and Savitzky–Golay (SG) filters was implemented to remove unwanted noise from the raw waveforms acquired by the electromagnetic flow sensors (FMG91-316L, Omega Engineering, Norwalk, CT, USA) and pressure transducers (PX309, Omega Engineering, Norwalk, CT, USA). Figure 8 shows the results of filtering the flow rate and pressure waveforms.

**Figure 9:**
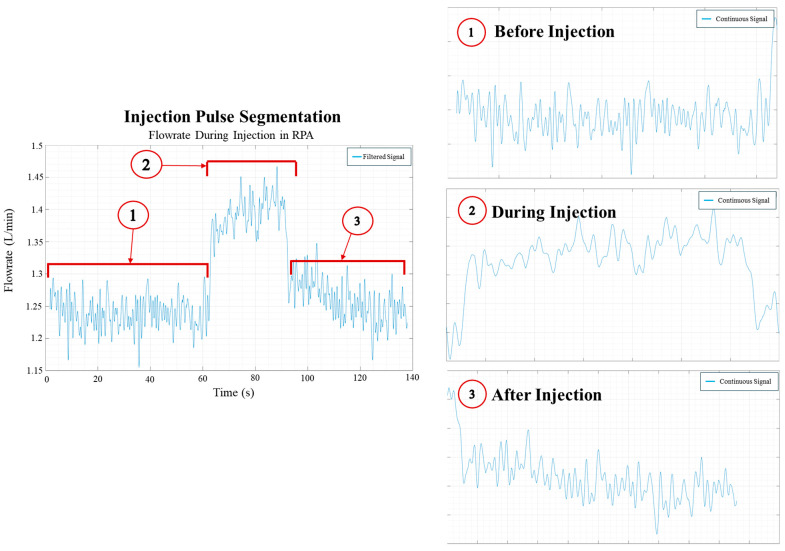
Segmentation of the injection process from flow rate data.

The filtered RPA flow waveforms (Q_RPA_) are segmented to extract the hemodynamic response of the injection sequence to the native flow field. These segmented temporal flow waveforms were further analyzed to quantify the jet entrainment rate and jet relaxation time. For each injection mode (continuous or pulsed), a filtered RPA flow waveform (Q_RPA_) is segmented into the following three stages, as shown in Figure 9.

i. Stage 1—Pre-injection period: This stage is used to acquire the native RPA flow waveform before the injections are sequenced.
ii. Stage 2—Active injection period: This stage captures the active injection period when the angiographic injection pump is engaged in the MFL through the RPA site for the predetermined interval, as mentioned in Table 1. In this stage, the jet rise time and settling time are calculated.
iii. Stage 3—Post-injection period: This stage captures the jet wind-down phase once the active injection period finishes. In this stage, the jet relaxation time is calculated.

**Figure 10:**
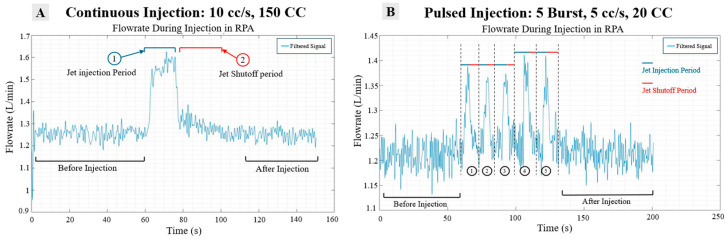
(**A**) Segmentation schematic of long shot injection and (**B**) short burst injection.

Furthermore, each active injection period (Stage 2) was characterized into two segments: (a) the jet injection period and (b) the jet shutoff period, as highlighted in Figure 10A. Since the pulsed protocols were carried out in a five-burst sequence, separate segmentations were performed for each jet burst (Figure 10B).

#### 2.4.2. In Vitro Entrainment and Jet Relaxation Quantification

As shown in Figure 7B, the key measurements in this study are the flow rates sampled at each distal PA branch, i.e., LPA and RPA, SVC, and IVC circulations. As previously discussed, the angiographic injector settings determine the volume injected as well as the volume flow rate per injection. This allows for a complete outlook of the flow field properties upon injection: before, during, and after.

To quantify the jet effect, two flow field properties were identified: entrainment and jet relaxation time. Given a co-flow configuration with a high-speed jet surrounded by a low-speed background flow, entrainment can be defined as the fluid transport across the fluid–fluid interface caused by shear-induced turbulence resulting in increased upstream flow. Flow entrainment rate ($Q_{ent}$) was quantified as

(3) $$Q_{ent}=Q_{meas,RPA}-\left(Q_{baseline,RPA}+Q_{inj}\right)\;$$

where $Q_{meas,RPA}$ is the measured flow rate distal to the injection site, $Q_{baseline,RPA}$ is the expected flow to RPA based on known $Q_{s}$ (systemic flow) and Pas flow split (set to 50-50), and $Q_{inj}$ is the arranged injection rate. Entrainment results in a positive $Q_{ent}$, whereas blockage would entail a negative $Q_{ent}$. Jet relaxation time is defined as the difference between jet rise time and fall time. Rise time can be understood as the time spanning from 10% to 90% of the flow field response to the injection; conversely, fall time is the reverse process as the jet winds down. We quantify the jet relaxation time $t_{rel}$ as

(4) $$t_{rel}=t_{rise}-t_{fall}\;$$

where $t_{rise}$ is the rise time and $t_{fall}$ is the fall time. Positive $t_{rel}$ indicates the low likelihood of downstream fluid buildup, as the co-flow takes longer to plateau than to dissipate the jet effect. Negative $t_{rel}$ would suggest a potential fluid buildup. Fluid buildup is an undesirable feature in a Fontan circulation model, where PVR is assumed to be constant. Jet relaxation time is a particularly powerful quantification in the case of pulsed injection (closely resembling real physiology), as it could highlight a potential fluid buildup that would cascade over many cycles.

#### 2.4.3. Jet Characterization

To gain a deeper understanding of jet behavior, it is essential to calculate both the entrainment rate and the relaxation time, as well as to analyze various response parameters extracted from the RPA flow rate signal. It is crucial to recognize that injections do not occur instantaneously; rather, there is a lag between the activation and stabilization of the jet within the RPA system. This stabilization process involves a spooling-up phase, during which the jet gradually reaches its peak flow rate at a specific peak time. After reaching this peak, the jet begins to stabilize, converging toward a steady settling value that persists throughout the duration of the activation.

**Figure 11:**
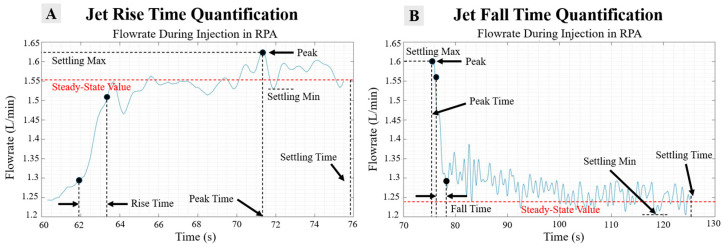
Jet rise time (**A**) and fall time (**B**) step response for the continuous jet at 10 cc/s, 150 cc.

The response dynamics of the flow field within the RPA are of paramount importance. One key indicator of the stability of the injection pulse is the settling time, which is defined as the duration required for the flow rate to drop within a 2% margin of the steady-state response values, as illustrated in Figure 11A,B. This metric serves as a critical benchmark for evaluating how effectively the injection stabilizes once it has been fully activated.

In addition to analyzing settling time, we assess the settling minimum and maximum values to quantify the extent of amplitude fluctuations in the jet response prior to achieving the steady-state condition. The calculation of the steady-state value is accomplished by determining both the overshoot and undershoot percentages of the jet response, which provides insight into how effectively momentum is transferred by the pulse and how promptly stability is attained within the system.

The data presented in Figure 11 represent the outcome of comprehensive post-processing applied to the RPA flow rate data from a long-duration injection case, specifically at a rate of 10 cc/s over a total volume of 150 cc. This post-processing includes thorough noise filtering and the segmentation of the flow rate signal into distinct activation and deactivation stages, allowing for targeted analysis of each phase. From these segmented signals, the peak value is first identified, followed by the determination of the settling value, which in turn facilitates the calculation of both rise time and fall time. Subsequently, the overall response of the flow field can be characterized by identifying the settling maximum and minimum values, as depicted in Figure 11A,B. Notably, Figure 11A indicates a peak overshoot occurring around t = 71 s.

For the pulsed injection sequences, the jet relaxation time alongside the associated response parameters is calculated for each of the five burst pulses within the injection sequence. These individual measurements are then averaged to provide a representative analysis of the entire sequence, enabling a more comprehensive understanding of the jet behavior under study.

## 3. Results

### 3.1. In Silico Flow Field

The ultimate purpose of the IJS is to reduce IVC pressure by exploiting the entrainment effect that transfers momentum from a high-speed jet to the low speed surrounding flow. Flow injectors that use the Bernoulli effect to entrain flow are not novel; however, in this case, given the application, there are physiological restrictions that prevent the model from (1) implementing the typical converging–diverging geometry found in common ejectors designs, (2) arbitrarily increasing jet speed or arbitrarily altering volume/mass flow, and (3) altering the entraining medium. SV physiology results in Fontan flow being purely passive and driven by the gradient between systemic venous return and pulmonary/atrial pressures. Highly efficient ejectors require a significant area ratio between the neck and the inlet to the converging–diverging section, a feature that would inevitably cause resistance in the Fontan conduit, resulting in further increase in pressure. The proposed IJS implementation shunting flow from the systemic circulation (aortic region) to the Fontan circulation operates on the arterial–venous pressure differential. Hence, the IJS effectiveness is restricted by the fixed range of time-dependent pressure gradients, which can be manipulated to a limited degree by altering the IJS diameter and nozzle shape. The entrainment mechanism itself leverages vortices and eddies to transfer momentum to the surrounding flow, which can be affected by the fluid properties.

The goal of this computational study is to understand and characterize the effective flow entrainment induced by the IJS, its effect on IVC flow and pressure, and co-flow dynamics. To achieve this, the proposed model isolates and combines flow sources (IVC and IJS) to then measure flow entrainment at the IVC boundary.

**Figure 12:**
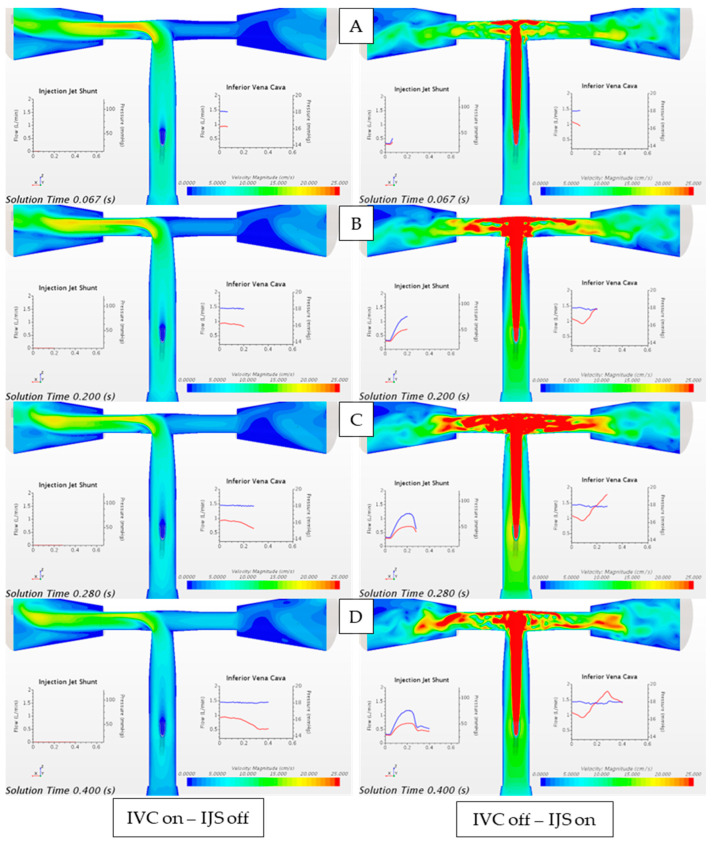
Velocity magnitude contour plot comparing the flow field when only IVC flow is instantiated through the unsteady volume force (IVC on, IJS off) and when only the IJS mass flow inlet is activated (IVC off, IJS on). Waveforms are reported over a single heart cycle at (**A**) early systole, (**B**) peak systole, (**C**) early diastole, and (**D**) late diastole. From plots (**A**–**D**): red-colored waveform represents volumetric flowrate and blue-colored waveform represents static pressure.

Figure 12 highlights the sharp difference in the flow field in the regional circulation when the IJS is actively shunting flow without any IVC volume force (Figure 12, right column) and when only IVC flow is instantiated (Figure 12, left column). It can be observed that IVC flow, being weakly pulsatile, offers a mostly uneventful smooth flow in the Fontan conduit, as expected. On the other hand, when the IJS is activated (in the absence of IVC), the dynamic transition the jet undergoes reflects on the Fontan conduit flow field. The nominal jet entrainment profile changes across the heart cycle. In systole, the jet offers significantly enhanced entrainment due to the laminar-to-turbulent transition, while in diastole, the jet entrainment effect weakens as the jet flow re-laminarizes. In Figure 12, it can be qualitatively observed that as the jet transitions from early systole to peak systole, the surrounding flow experiences a strong radial entrainment that results in the jet cone growth nearly obstructing the TCPC connection along the nozzle major axis. As the jet transitions back to diastole entrainment, the radial “pull” is weakened, and the jet cone regresses.

**Figure 13:**
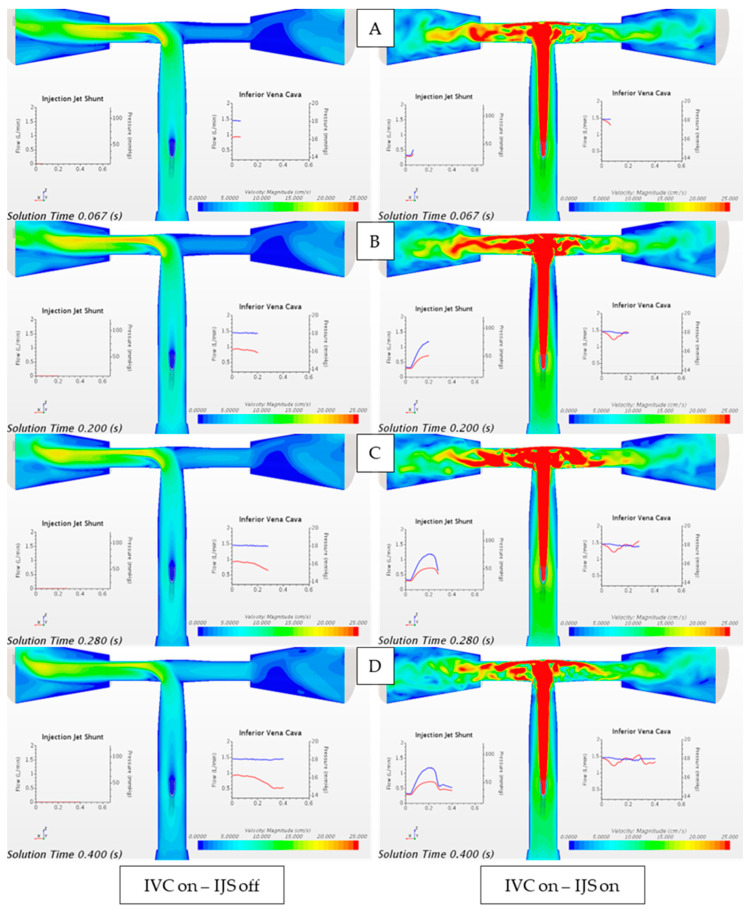
Velocity magnitude contour plot comparing the flow field when only IVC flow is instantiated through the unsteady volume force (IVC on, IJS off) and when superimposed to the active IJS mass flow inlet (IVC on, IJS on). Waveforms are reported over a single heart cycle at (**A**) early systole, (**B**) peak systole, (**C**) early diastole, and (**D**) late diastole. From plots (**A**–**D**): red-colored waveform represents volumetric flowrate, and blue-colored waveform represents static pressure.

Figure 13 compares the case where only IVC flow is instantiated with the case when the IJS is activated alongside the IVC volume force. This comparison represents a step forward from the model shown in Figure 12. While in Figure 12, the IJS nominal entrainment capacity is isolated, the model shown in Figure 13 offers insight into how the IJS behaves when co-flow is present. In this case, the IJS is still capable of a significant entrainment, however weakened from the nominal entrainment. This is probably due to the finite volume that the native co-flow (IVC flow) already occupies as the IJS injects flow into the Fontan conduit, thereby limiting the additional peak entrainment shown in Figure 12C,D. Similar to Figure 12, Figure 13 displays the transitional effect of the jet across a single heart cycle as well as the growth in the cone as the jet transitions from diastole to systole.

**Figure 14:**
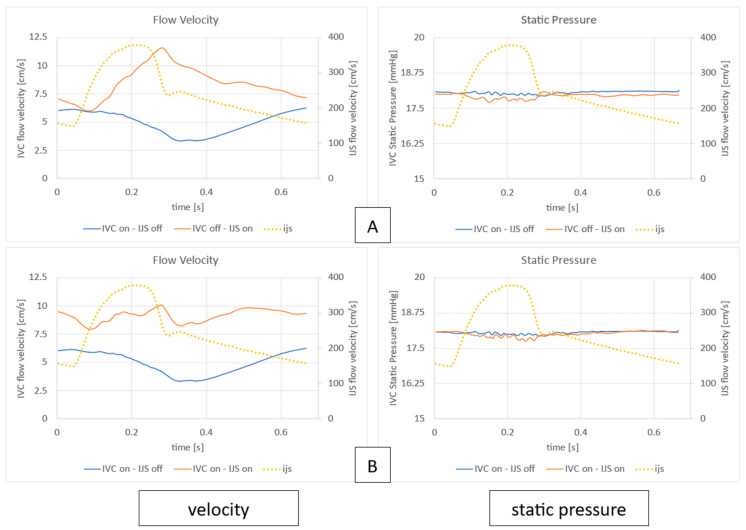
Flow field measurements comparing IVC static pressure and average IVC flow velocity when (**A**) IVC flow is instantiated through the unsteady volume force (IVC on, IJS off) with the IJS mass flow inlet activated alone (IVC off, IJS on), and (**B**) when IVC flow is instantiated through the unsteady volume force (IVC on, IJS off) compared to the case where IVC flow is superimposed to the active IJS mass flow inlet (IVC on, IJS on).

In Figure 14, the qualitative overview of the flow field offered in Figure 12 and Figure 13 are quantified. Here, static pressure and IVC flow velocity are reviewed to highlight entrainment differences between the nominal IJS effect and the IJS effect with co-flow. In Figure 12, the focus shifts on the relative flow velocities when the IJS has been activated. The goal here is to observe how effectively momentum is transferred. In each case (IVC off, IJS on and IVC on, IJS on), it can be readily observed that while the IJS can reach velocities as high as 380 cm/s at peak systole, the surrounding flow experiences acceleration from a stagnant state but cannot reach even 5% of the jet’s top speed (10–12 cm/s). This outcome is due to the poor efficiency of momentum transfer related to the small volume flow rate emerging from the IJS, the duration of effective entrainment (occurring only in systole), and the absence of the typical ejector geometry that would enhance entrainment. When only the IJS is active, the surrounding flow reaches an average velocity of 8.49 cm/s and a peak of 11.60 cm/s. The superposition of IJS flow and IVC flow results in an average velocity of 9.15 cm/s and a peak of 10.08 cm/s. In addition, comparing the nominal case to the co-flow case across a single heart cycle, it can be seen that (1) as the IJS flow injection increases, IVC flow initially drops; (2) then, as IJS effective regime reaches its peak, positive entrainment occurs as IVC flow increases; (3) as the IJS effective regime ends, IVC flow suffers a reduction; and (4) lastly, as the IJS flow injection further drops, the IVC flow recovers. This entrainment sequence can once again be attributed to the tradeoff of the finite volume available to co-flow and IJS flow. The static pressure plots in Figure 13 complete the picture by showing the effects on IVC pressure due to the IJS activation, comparing the nominal entrainment and the IJS entrainment with the co-flow. Overall, given the low momentum transfer shown, the effect on IVC pressure (<1 mmHg) confirms low efficiency across the heart cycle. It must be noted, however, that the effect on systole is larger than that on diastole. While the drop in IVC pressure is similar in diastole, in systole the transition from the nominal model to the co-flow model results in a 40% reduction in pressure drop.

### 3.2. In Silico Entrainment Analysis

**Figure 15:**
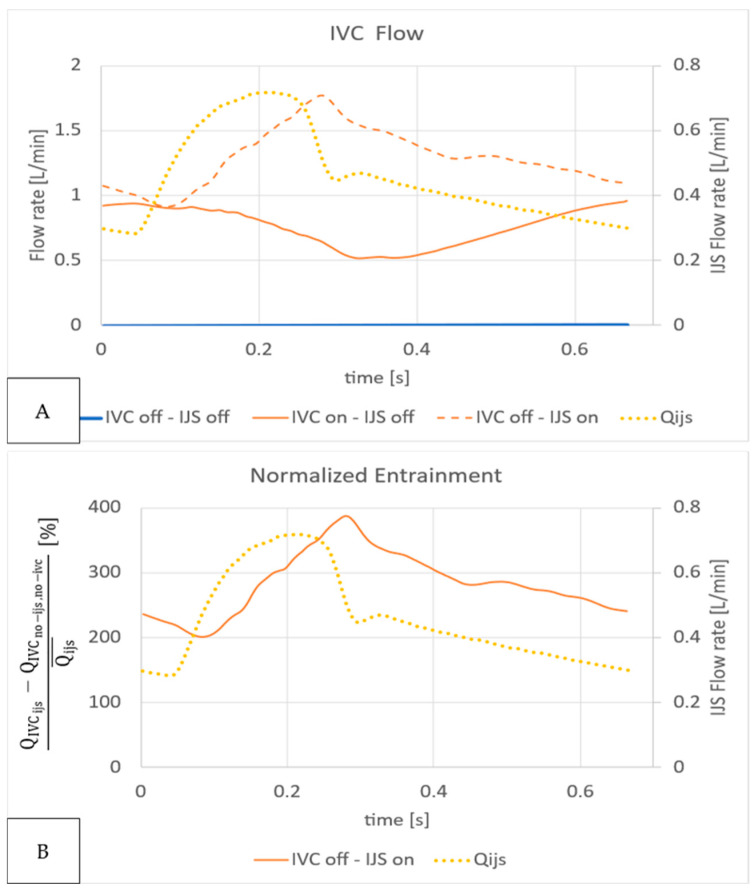
IVC flow entrainment quantification of IJS implementation. (**A**) IVC volume flow rate $Q_{IVC}$ without IVC volume force or IJS (blue), with IJS only (dotted orange), and with IVC volume force only (solid orange) overlaid to IJS volume flow rate $Q_{ijs}$ (dotted yellow). (**B**) The relative change in IVC flow ($Q_{IVC_{ijs}}-Q_{IVC_{no-ijs,no-ivc}}$) normalized to the cycle average IJS volume flow rate $\bar{Q_{ijs}}$ (orange curve) overlaid to IJS volume flow rate $Q_{ijs}$ (dotted yellow curve).

**Figure 16:**
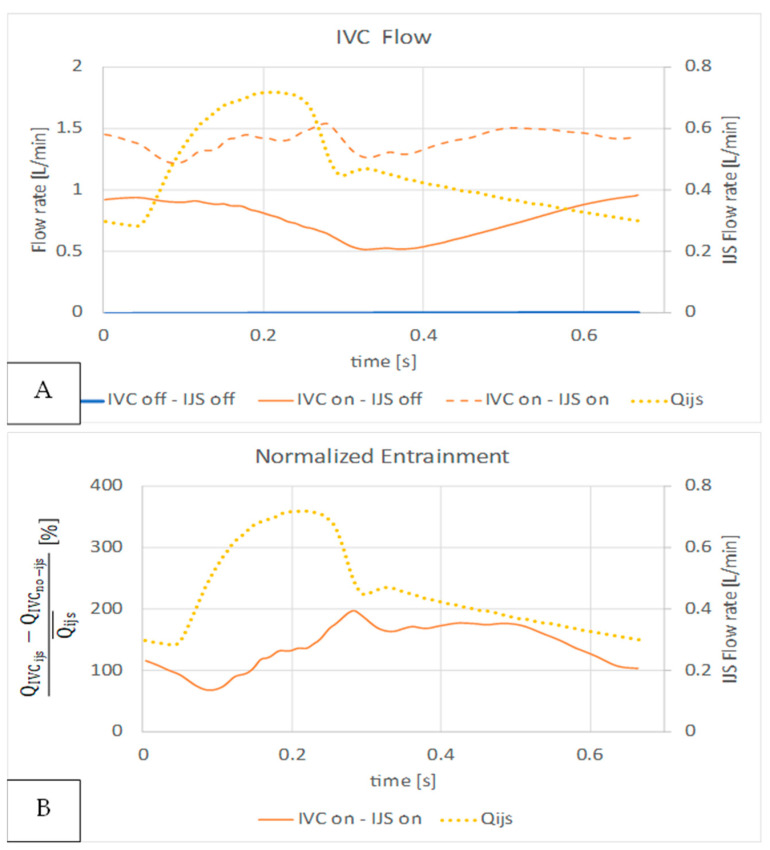
IVC flow entrainment quantification of IJS implementation. (**A**) IVC volume flow rate $Q_{IVC}$ without IVC volume force or IJS (blue), with IVC volume force only (solid orange), and with IJS and IVC volume force (dotted orange) overlaid to IJS volume flow rate $Q_{ijs}$ (dotted yellow). (**B**) The relative change in IVC flow ($Q_{IVC_{ijs}}-Q_{IVC_{no-ijs}}$) normalized to the cycle average IJS volume flow rate $\bar{Q_{ijs}}$ (orange curve) overlaid to IJS volume flow rate $Q_{ijs}$ (dotted yellow curve).

The IJS contribution to IVC flow entrainment is entirely predicated on efficacious momentum transfer from the high-speed jet to the low-speed environmental flow. Entrainment was therefore quantified as a function of time over the cardiac cycle based on the effective entrainment calculated as the relative difference between IVC flow rates normalized by the cycle-averaged IJS flow rate $\bar{Q_{\mathrm{ijs}}}$ shown in Figure 15 and Figure 16. We compare IVC flow rates with ($Q_{{\mathrm{IVC}}_{\mathrm{ijs}}}$) and without ($Q_{{\mathrm{IVC}}_{\mathrm{no}-\mathrm{ijs}}}$) the shunt in absence of IVC flow, overlaid to the IJS flow rate in Figure 15A. Qualitatively, the transient nature of entrainment is highlighted by the fact that while in systole, when IJS flow is at its peak, IVC flow increases, and during diastole, the IJS insertion results in a reduction of IVC flow as expected. Figure 15 highlights the jet’s significant ability to entrain flow. Quantitatively, the normalized entrainment rate calculated as $\left(Q_{{\mathrm{IVC}}_{\mathrm{ijs}}}-Q_{{\mathrm{IVC}}_{\mathrm{no}-\mathrm{ijs},\;\mathrm{no}-\mathrm{ivc}}}\right)/\bar{Q_{\mathrm{ijs}}}$, shown in Figure 15B, can be as high as 387% (in systole) and as low as 200% (in diastole), indicating a notable difference across the cardiac cycle.

In Figure 16, we compare IVC flow rates with ($Q_{{\mathrm{IVC}}_{\mathrm{ijs}}}$) and without ($Q_{{\mathrm{IVC}}_{\mathrm{no}-\mathrm{ijs}}}$) the shunt with IVC co-flow, overlaid to the IJS flow rate in Figure 16A. As previously observed, the transient nature of entrainment is highlighted as the IJS transitions to its peak regime, resulting in first a reduction in IVC flow and then an increase. In diastole, the IJS insertion results in first a reduction of IVC flow and then a recovery. Figure 15A highlights the jet’s significant ability to entrain IVC flow as well as the relative tradeoff in the available volume between IJS flow and IVC flow. Quantitatively, the normalized entrainment rate, calculated as $\left(Q_{{\mathrm{IVC}}_{\mathrm{ijs}}}-Q_{{\mathrm{IVC}}_{\mathrm{no}-\mathrm{ijs}}}\right)/\bar{Q_{\mathrm{ijs}}}$ and shown in Figure 16B, can be as high as 197% (in systole) and as low as 69% (in diastole), indicating a significant difference across the cardiac cycle and with respect to a model lacking co-flow.

### 3.3. In Vitro Flow Field

**Figure 17:**
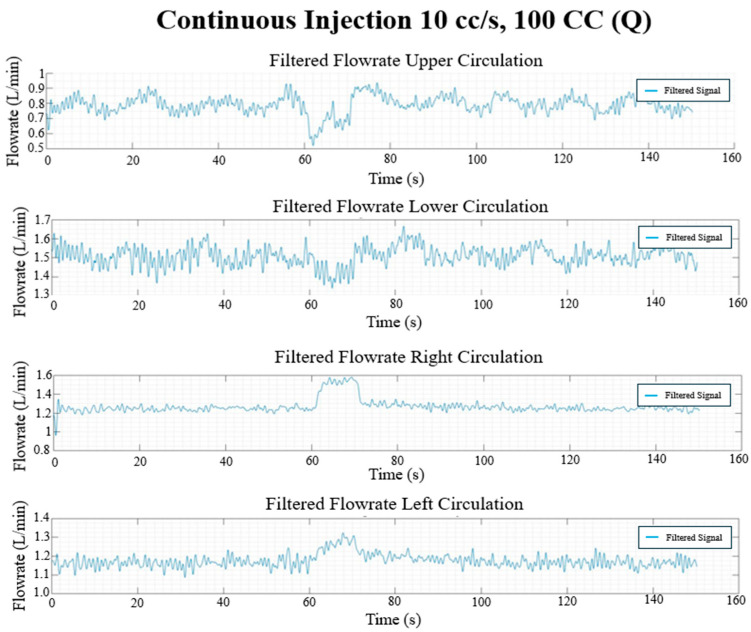
Continuous injection flow rate waveforms (L/min) in each circulation branch for 10 cc/s injection rate and 100 CC injection volume.

**Figure 18:**
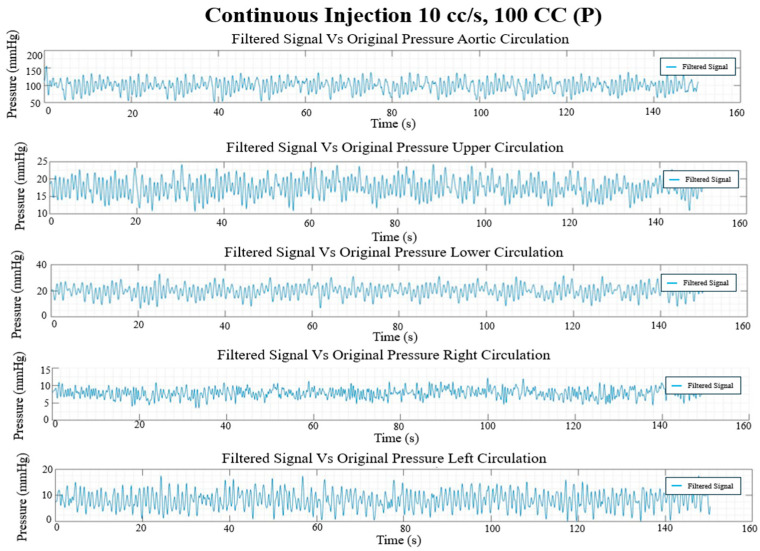
Continuous injection pressure waveforms (mmHg) in each circulation branch for 10 cc/s injection rate and 100 CC injection volume.

**Figure 19:**
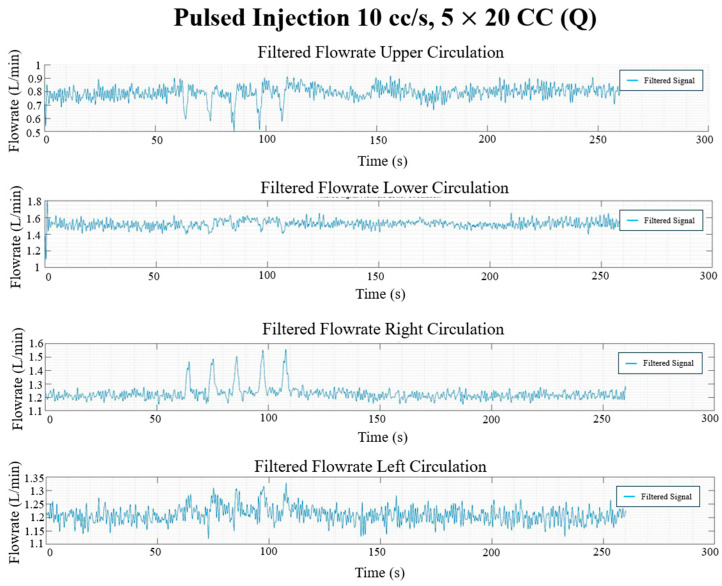
Pulsed injection flow rate waveforms (L/min) in each circulation branch for 10 cc/s injection rate and 5 × 20 CC burst volume.

**Figure 20:**
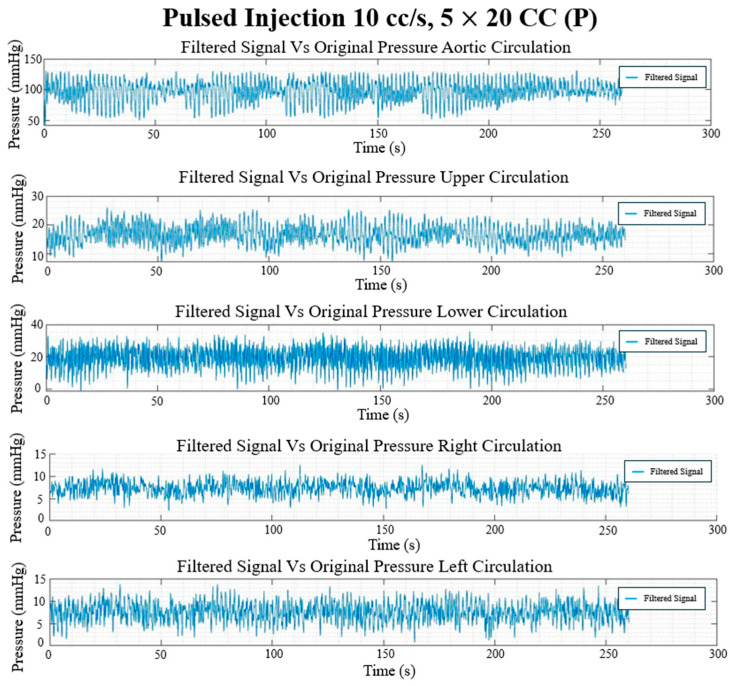
Pulsed injection pressure waveforms (mmHg) in each circulation branch for 10 cc/s injection rate and 5 × 20 CC burst volume.

The final filtered result of the pressure and flow rate for the long, continuous injection at 10 cc/s and 100 cc injection volume and the pulsed injection at 10 cc/s and 5 × 20 cc injection volume cases are given in Figure 17, Figure 18, Figure 19 and Figure 20, showing the hemodynamic response of the MFL. The pressure waveform for each branch is observed to be constant throughout the injection process, indicating that the injection protocol does not alter the pressure across the PAs. The flow waveforms for each branch show more prominent responses to the injections.

Each experimental run from Table 1 was performed for three trials (*n* = 3). Once the experimental protocol has been completed, flow rates and pressure sampled at described locations were cycle-averaged and are included in Table 2 and Table 3, respectively.

#### Detailed Jet Effect Analysis

**Figure 21:**
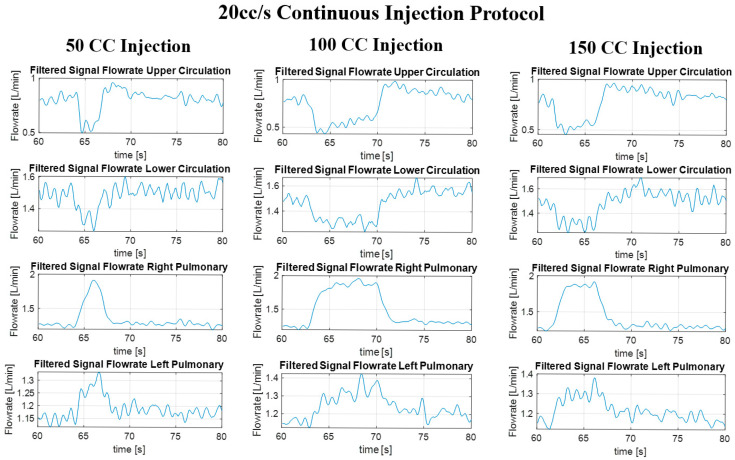
Transient hemodynamic redistribution in TCPC branches during continuous injection at varying volumes. Overlay of flow rate waveforms in right and left pulmonary branches (RPA and LPA), and upper and lower systemic branches (SVC and IVC) during continuous jet injections at 20 cc/s for increasing injection volumes (50 cc, 100 cc, 150 cc).

As previously observed, while the pressure waveforms did not show significant fluctuations in response to injections across the protocols, the flow rate waveforms feature more interesting dynamics. RPA flowrate increases as the injection occurs, as expected. However, in the other branches, the effects are different (Figure 21). Upon injection, the upstream flow rates (upper and lower systemic) experience a sudden decrease. Following this reduction in the flow rate and for the duration of the injection, the upper circulation experiences a gradual increase in flow rate, whereas the lower circulation does not show a prominent response. This response mismatch between upper and lower circulation can be associated with the flow split ratio, as outlined in Section 2.4. Concurrently, the LPA waveform also experiences a mild increase, which appears to match the localized flow increase in the upper circulation. These flow dynamics were consistently observed across all injection protocols. The jet effects are enhanced with increasing injection flowrate and prolonged injection volume (Figure 21).

A trend similar to the one discussed above can be seen in Figure 16A for the CFD results. In the early systole, the IVC flow rate decreases in response to the IJS flow increase. As the jet transitions to the peak systole, the IVC flow rate recovers. Magnitude changes in the flow rate responses are also measurably similar, as shown in Figure 14A and Figure 20.

### 3.4. In Vitro Entrainment Analysis

Once the experimental protocol has been completed, flow rates sampled at the described locations were time averaged. Entrainment was calculated using Equation (3) for the whole parameter space tested in Table 1, and the results are tabulated in Table 4 and Table 5 for the continuous and pulsed experimental runs. Green values ($Q_{ent}$ > 0) represent cases for which entrainment was detected, and red values ($Q_{ent}$ < 0) are cases where blockage occurred.

One can observe how, for either continuous or pulsed injections, varying the total volume injected has no significant influence on the entrainment rate across all injection rates. On the other hand, for both injection conditions, increasing the injection rate results in a reduction in entrainment that is consistent across all injected volumes. Given the constant diameter shunt (7 Fr) used in this experiment, this outcome suggests that entrainment is strongly correlated to the co-flow velocity ratio (and flow rate ratio) and weakly correlated to the total injected volume. In addition, Table 4 and Table 5 offer significant insight into the differences between injection conditions (continuous vs. pulsed). In Table 4, the entrainment cutoff appears to be $Q_{inj}\;$~0.6 L/min, whereas in Table 5 the cutoff occurs from a smaller flow rate, $Q_{inj}$~0.3 L/min. To better evaluate the degree of entrainment, the tabulated maximum values (for $Q_{inj}$ 0.3) can be normalized by the $Q_{inj}$ of the specific case. After averaging across the column, $Q_{ent}/Q_{inj}\;$= 0.63 for a continuous injection, and $Q_{ent}/Q_{inj}$ = 0.16 for a pulsed injection. This stark contrast highlights a clear difference between injection conditions, which emphasize the importance of ensuring proper physiological modeling: pulsatility cannot be overlooked. Conversely, this opens the door for a discussion involving pulsatility modulation to enhance entrainment. Since the shunt diameter is kept constant, an increasing flow rate indicates an increased co-flow velocity ratio ($\upsilon_{jet}/\upsilon_{background}$), and it would sound counterintuitive to have increasingly positive results for a decreasing velocity ratio. However, the results presented in the following section for the jet relaxation time will provide further insight into the jet dynamics and clarify this puzzling outcome.

### 3.5. In Vitro Jet Relaxation Analysis

Table 6 and Table 7 offer an overview of the calculated jet relaxation time $t_{rel}$ for the same parameter space explored in the previous section. Green values ($t_{rel}$ > 0) represent a lack of potential fluid buildup, and red values ($t_{rel}$ < 0) indicate a high likelihood of fluid buildup. Due to the choice of sampling rate in the experimental phase, the tabulated observations may show some inconsistency; nevertheless, in most cases, a trend can be perceived for relaxation time.

In Table 6, aside from the data reported for $V_{inj}$ = 0.1, which displays an opposite tendency, it can be observed that varying the injected volume $V_{inj}$ does not significantly alter $t_{rel}$. On the other hand, increasing $Q_{inj}$ appears to display a decreasing trend, with a maximum for $Q_{inj}=0.6$. This would indicate that for increasing the injection flow rate, the fall time tends towards the rise time, suggesting that for increasing the co-flow velocity ratio, the flow field requires significantly more time to dissipate the jet effect. In Table 7, a similar trend can be seen for pulsed injections. Such an outcome is expected. This flow relaxation feature helps understand the counterintuitive observation made for the entrainment quantifications where enhanced entrainment is registered for a lower co-flow velocity ratio. These observations become even more relevant for the pulsed injection cases.

**Figure 22:**
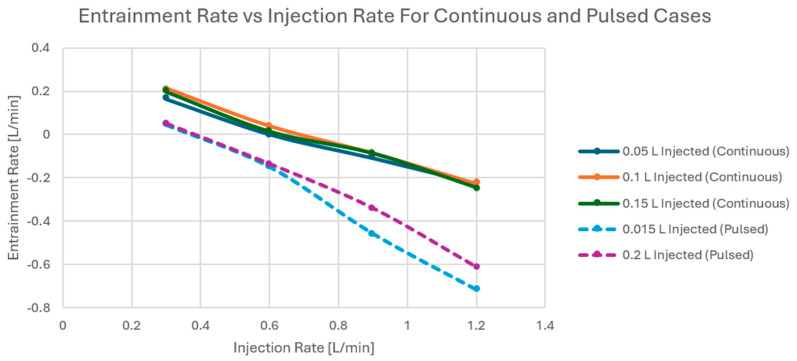
Continuous injection entrainment rate versus $Q_{inj}$ at $V_{inj}$ = 0.05 L, 0.1 L, 0.15 L.

From a physiological standpoint, the jet effect is constrained by the ratio of cardiac output to systemic flow, which expresses the amount of shunted flow. Injection shunt hemodynamics are influenced by shunt size and pressure gradient across the shunt. As observed in the above results, the effects of entrainment are reliant upon the complex interplay of injection speed, injection volume, and pulsatility, as shown in Figure 22.

A similar trend has been seen for pulsed injections, where entrainment decreased with the increase in injection rate. Notably, this trend is reduced with pulsed injections.

A different trend is observed when looking at the jet relaxation time of the individual pulses.

## 4. Discussion

In recent work detailed in several reports, injection jets have gained interest and potential in the field of palliative care to address problematic physiologies in single-ventricle patients. The goals of this computational study were to (1) generate models able to isolate the IJS ability to entrain IVC flow, (2) qualitatively and quantitatively evaluate IVC flow dynamics, and (3) estimate entrainment. It was shown that the IJS can entrain a significant amount of flow; however, the momentum transfer from the high-speed jet to the surrounding flow is not efficient and results in weak surrounding flow acceleration and low IVC pressure drop. Moreover, while the IJS has a large peak entrainment in the absence of co-flow, entrainment weakens with co-flow due to the transient finite conduit volume tradeoff between the injected flow and IVC flow. It must be noted that on top of these observations, the IJS effectiveness is strongly coupled to the heart cycle phase. During systole, the IJS is most efficient, and in the presence of co-flow, its efficiency is further affected by the volume tradeoff, whereas during diastole, the momentum transfer is significantly reduced as expected.

The goal of this in vitro study was to quantify the degree and dynamics of entrainment by quantifying (1) the amount of entrained fluid and (2) the relaxation of the jet with respect to injection rate and total injected volume. IJS-induced volume buildup can occur in two modalities. For a fixed volume, as the injection rate increases, the additional volume cannot be accommodated, thereby effectively obstructing entrainment. Alternatively, pulsed jets may suffer from a transient volume buildup, as the injected volume has not been dissipating fast enough between consecutive pulses. It was demonstrated that for increasing injection rate, the observed entrainment effect was reduced for any injected volume. This trend was preserved for both continuous and pulsed injection modalities. The measured relaxation times elucidate the fact that the decreasing trend in entrainment for increased injection rates cannot be attributed to a volume buildup phenomenon associated with jet dissipation. This was clearly shown in the pulsed jet results, where no negative relaxation times were detected. This indicates that the inability to entrain the flow can only be caused by the conduit size where the shunt is inserted.

In silico and in vitro results have successfully demonstrated and quantified the entrainment effect of the IJS. While the jet is capable of entraining a significant amount of flow, the resultant momentum transfer does not produce a significant drop in upstream pressure. Furthermore, the entrainment effect is transient and dependent on the injection rate. In systole, entrainment is maximized, while in diastole, the jet may obstruct the co-flow. The most concerning phenomenon is the jet-related volume buildup; fortunately, this has been addressed in our ongoing work by introducing a fenestration. In Fontan patients, fenestration, which is usually an orifice resulting in a right to left flow between the Fontan conduit and the right atrium, effectively drops the venous pressure. In our ongoing effort, the insertion of the fenestration is designed to assist the venous pressure reduction and dissipate the injection volume.

## 5. Limitations

The in vitro study has two notable limitations. The data acquisition system, utilizing NI DAQ cards (Models 9361 and 9205), operates with constraints on sampling frequency and temporal resolution, which may introduce minor inaccuracies in capturing transient hemodynamic responses to calculate the jet relaxation time for the pulsed cases. Additionally, FMG91 series electromagnetic flow sensors were used for the flow field measurements. While these sensors acquire very accurate temporal waveforms, they do not offer spatial resolution, which is critical for a detailed analysis of entrainment dynamics. Future efforts could benefit from employing higher-frequency sampling techniques and advanced optical techniques, such as particle image velocimetry (PIV), for improved spatial and temporal insights. Furthermore, the geometric model employed in this benchtop study is different from our newer conception of the location of the IJS nozzle, which more resembles the in silico model shown.

The in silico study employs an unsteady volume force to simulate the injection jet shunt (IJS) effect. While this approach effectively isolates the positive momentum transfer due to the entraining jet, it simplifies the complex flow interactions that could be more comprehensively captured with detailed boundary conditions by utilizing the full-scale LPM, as performed in our previous studies.

## 6. Conclusions

We conducted both computational and experimental analyses of the complex dynamics of the IJS co-flow in the Fontan conduit. Our proposed approach, which involves IJS-assisted Fontan circulation, challenges the existing paradigm that a mechanical pump is necessary for actively assisting the Fontan circulation. If successful, an optimized IJS design integrated into a fenestrated Fontan conduit could address significant barriers in this field by providing a novel and effective alternative for palliation in failing Fontan patients. This approach has the potential to delay or even eliminate the need for heart transplantation or the implantation of mechanical pumps, which are associated with well-known complications.

## Figures and Tables

**Table 1:** Injection protocol parameter space.

	Injections					
Case #	Type	Flow Rate		Number (#)	Size (cc)	Total Volume (L)
		(cc/s)	(L/min)			
	Short Multiple Injection					
1		5	0.3	5	20	0.100
2		5	0.3	5	15	0.075
3		10	0.6	5	20	0.100
4		10	0.6	5	15	0.075
5		15	0.9	5	20	0.100
6		15	0.9	5	15	0.075
7		20	1.2	5	20	0.100
8		20	1.2	5	15	0.075
	Long Single Injection					
9		5	0.3	1	50	0.050
10		5	0.3	1	100	0.100
11		5	0.3	1	150	0.150
12		10	0.6	1	50	0.050
13		10	0.6	1	100	0.100
14		10	0.6	1	150	0.150
15		15	0.9	1	50	0.050
16		15	0.9	1	100	0.100
17		15	0.9	1	150	0.150
18		20	1.2	1	50	0.050
19		20	1.2	1	100	0.100
20		20	1.2	1	150	0.150

**Table 2:** In vitro flow rate results.

Case	Flow Rate (L/min)			
	$Q_{upper}$	$Q_{lower}$	$Q_{RPA}$	$Q_{LPA}$
1	16.56	18.77	7.27	7.90
2	16.57	18.76	7.31	7.54
3	16.56	18.81	7.26	7.59
4	16.68	18.86	7.37	7.58
5	16.99	19.21	7.52	8.43
6	17.41	19.55	7.66	8.94
7	17.60	19.75	7.70	8.94
8	17.42	19.63	7.65	8.93
9	17.36	19.56	7.70	8.23
10	17.58	19.74	7.78	8.32
11	17.55	19.74	7.73	8.31
12	17.41	19.61	7.69	8.26
13	17.59	19.83	7.79	8.24
14	17.78	20.01	7.73	8.29
15	17.28	19.48	7.75	8.22
16	17.27	19.51	7.63	8.17
17	17.36	19.59	7.79	8.20
18	17.39	19.45	7.64	8.99
19	17.42	19.61	7.61	8.89
20	17.46	19.61	7.66	8.98

**Table 3:** In vitro pressure results.

Case	Pressure (mmHg)				
	$P_{upper}$	$P_{lower}$	$P_{RPA}$	$P_{LPA}$	$P_{aortic}$
1	16.56	18.77	7.27	7.90	94.19
2	16.57	18.76	7.31	7.54	98.31
3	16.56	18.81	7.26	7.59	97.95
4	16.68	18.86	7.37	7.58	98.41
5	16.99	19.21	7.52	8.43	96.88
6	17.41	19.55	7.66	8.94	96.80
7	17.60	19.75	7.70	8.94	98.02
8	17.42	19.63	7.65	8.93	98.14
9	17.36	19.56	7.70	8.23	97.39
10	17.58	19.74	7.78	8.32	97.39
11	17.55	19.74	7.73	8.31	97.31
12	17.41	19.61	7.69	8.26	98.38
13	17.59	19.83	7.79	8.24	98.50
14	17.78	20.01	7.73	8.29	99.25
15	17.28	19.48	7.75	8.22	98.70
16	17.27	19.51	7.63	8.17	98.27
17	17.36	19.59	7.79	8.20	97.96
18	17.39	19.45	7.64	8.99	97.36
19	17.42	19.61	7.61	8.89	96.87
20	17.46	19.61	7.66	8.98	96.77

**Table 4:** Entrainment flow rate $Q_{ent}$ [L/min] for various injection rates $Q_{inj}$ and injection volumes $V_{inj}$ for continuous injections.

		$Q_{inj}\;\left[L/min\right]$			
		0.3	0.6	0.9	1.2
	**0.05**	0.1646	0.0015	−0.1084	−0.2263
$V_{inj}\;\left[L\right]$	**0.1**	0.2125	0.0389	−0.086	−0.2265
	**0.15**	0.1985	0.0144	−0.0866	−0.2454

**Table 5:** Entrainment flow rate $Q_{ent}$ [L/min] for various injection rates $Q_{inj}$ and injection volumes $V_{inj}$ for pulsed injections.

		$Q_{inj}\;\left[L/min\right]$			
		0.3	0.6	0.9	1.2
$V_{inj}\;\left[L\right]$	**0.015**	0.04412	−0.14936	−0.4581	−0.7167
	**0.2**	0.04964	−0.1353	−0.33948	−0.60956

**Table 6:** Jet relaxation time [s] for various injection rates $Q_{inj}$ and injection volumes $V_{inj}$ for continuous injections.

		$Q_{inj}\;\left[L/min\right]$			
		0.3	0.6	0.9	1.2
	**0.05**	0.497	0.891	0.398	0.198
$V_{inj}\;\left[L\right]$	**0.1**	0.103	0.100	0.509	0.504
	**0.15**	0.495	0.607	0.399	0.107

**Table 7:** Jet relaxation time [s] for various injection rates $Q_{inj}$ and injection volumes $V_{inj}$ for pulsed injections (5 pulses).

		$Q_{inj}\;\left[L/min\right]$			
		0.3	0.6	0.9	1.2
$V_{inj}\;\left[L\right]$	**0.015**	0.715	0.341	0.118	0.151
	**0.2**	0.196	0.315	0.809	0.021

## Data Availability

All data generated or analyzed during this study are included in this published article.

## Appendix A

**Table A1:** List of acronyms.

Single ventricle	SV
Hypoplastic left heart syndrome	HLHS
Computational fluid dynamics	CFD
Mock flow loop	MFL
Ventricular assist device	VAD
Injection jet shunt	IJS
Superior vena cava	SVC
Inferior vena cava	IVC
Left pulmonary artery	LPA
Right pulmonary artery	RPA
Lumped parameter model	LPM
Body surface area	BSA
Pulmonary artery	PA
Expanded polytetrafluoroethylene	ePTFE
American Heart Association	AHA
Resistor	R
Capacitor	C
Inductor	L
Boundary condition	BC
Wall-adapted local eddy-viscosity	WALE
Backwards differential formulation	BDF
Cardiac output	CO
Systemic flow	Qs
Flow rate	Q
Beats per minute	BPM
Multichannel data acquisition	DAQ
Savitzky–Golay	SG
Flow entrainment rate	$Q_{ent}$
Rise time	$t_{rise}$
Fall time	$t_{fall}$
Relaxation time	$t_{rel}$

**Table A2:** Nomenclature and location of LPM elements for each compartment in the in vitro study.

MFL Setup	Resistor	Compliance	Flow Sensor	Pressure Sensor
Upper Systemic LPM Compartment	R_UPPER SYS_	C_UPPER SYS_	Q_UPPER SYS_	P_UPPER SYS_
Lower Systemic LPM Compartment	R_LOWER SYS_	C_LOWER SYS_	Q_LOWER SYS_	P_LOWER SYS_
Left Pulmonary LPM Compartment	R_LPA_	C_LPA_	Q_LPA_	P_LPA_
Right Pulmonary LPM Compartment	R_RPA_	C_RPA_	Q_RPA_	P_RPA_
